# The Signaling Role of CD40 Ligand in Platelet Biology and in Platelet Component Transfusion

**DOI:** 10.3390/ijms151222342

**Published:** 2014-12-03

**Authors:** Chaker Aoui, Antoine Prigent, Caroline Sut, Sofiane Tariket, Hind Hamzeh-Cognasse, Bruno Pozzetto, Yolande Richard, Fabrice Cognasse, Sandrine Laradi, Olivier Garraud

**Affiliations:** 1 Immunity of Mucosa and Pathogen Agents Group (GIMAP-EA3064), University of Lyon, Saint-Etienne 42023, France; E-Mails: antoine.prigent@efs.sante.fr (A.P.); caroline-sut@hotmail.fr (C.S.); tariket.sofiane@hotmail.fr (S.T.); hind.hamzeh@univ-st-etienne.fr (H.H-C.); bruno.pozzetto@univ-st-etienne.fr (B.P.); sandrine.laradi@efs.sante.fr (S.L.); ogarraud@ints.fr (O.G.); 2 French Blood Establishment, EFS Auvergne-Loire, Saint-Etienne 42023, France; 3 INSERMu1016, Institut Cochin, Departement “Infection, Immunity and Inflammation”, Paris 75014, France; E-Mail: Yolande.richard@inserm.fr; 4 CNRS-UMR8104, Cochin Institute, Paris 75014, France; 5 Université Paris-Descartes, Sorbonne Paris Cité, Paris 75270, France; 6 Institut National de Transfusion Sanguine (INTS), Paris 75739, France

**Keywords:** CD40 ligand, CD40, inflammation, signaling pathways, p38 mitogen-activated protein kinases (MAPK), nuclear factor-KappaB (NF-κB)

## Abstract

The CD40 ligand (CD40L) is a transmembrane molecule of crucial interest in cell signaling in innate and adaptive immunity. It is expressed by a variety of cells, but mainly by activated T-lymphocytes and platelets. CD40L may be cleaved into a soluble form (sCD40L) that has a cytokine-like activity. Both forms bind to several receptors, including CD40. This interaction is necessary for the antigen specific immune response. Furthermore, CD40L and sCD40L are involved in inflammation and a panoply of immune related and vascular pathologies. Soluble CD40L is primarily produced by platelets after activation, degranulation and cleavage, which may present a problem for transfusion. Soluble CD40L is involved in adverse transfusion events including transfusion related acute lung injury (TRALI). Although platelet storage designed for transfusion occurs in sterile conditions, platelets are activated and release sCD40L without known agonists. Recently, proteomic studies identified signaling pathways activated in platelet concentrates. Soluble CD40L is a good candidate for platelet activation in an auto-amplification loop. In this review, we describe the immunomodulatory role of CD40L in physiological and pathological conditions. We will focus on the main signaling pathways activated by CD40L after binding to its different receptors.

## 1. Introduction

CD40 ligand (CD40L)—otherwise known as CD154—is of particular interest for several reasons. It is easily detectable in plasma; it is essential to immunity at large and central to adaptive immunity, being among the seminal molecules that tether antigen (Ag)-specific T and B-lymphocytes in the synapse; and it is indispensable for the formation of germinal centers (GCs) in lymph nodes [1,2,3,4]. CD40L is thus crucial for cell signaling in both adaptive and innate immunity, as it is expressed by a large variety of cells that take a role in immune responses [1,4]. Further, CD40L has genetic and molecular polymorphisms, with pathogenic and pathologic consequences [5]. Intriguingly, its soluble form is principally generated by platelets, and it is responsible for transfusion associated hazards [6,7,8]. Together, those properties require the attention of pathologists and clinicians, as CD40L is more important in medicine than initially thought.

In this review, we will discuss the role of CD40L and its soluble form (sCD40L) in transfusion hazards. It is associated with high levels of inflammatory molecules such as chemokines, cytokines and biological response modifiers (BRMs) released by platelets during storage. sCD40L is a master pro-inflammatory BRM in transfusion [6,7,8,9,10,11,12]. Platelet sCD40L has been largely studied in inflammation and autoimmune disease [3,13,14,15], but the mechanism for its regulation is just beginning to be unraveled.

## 2. What Is CD40L?

**Figure 1:**
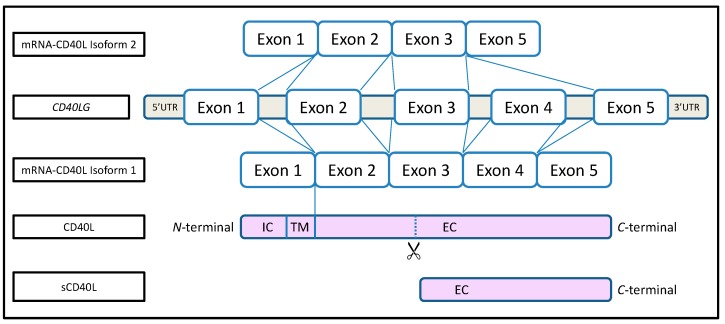
Sheme of the CD40 ligand gene structure and its different isoforms. Intracellular domain (IC), transmembrane domain (TM), extracellular domain (EC).

CD40L is a 33 kDa type II transmembrane protein belonging to the Tumor Necrosis Factor (TNF) superfamily. The CD40L gene (*CD40LG*) encodes a 261 amino acid (AA) protein with a 22 AA cytoplasmic domain, a 24 AA transmembrane (TM) domain, and a 215 AA extracellular domain (Figure 1) [3]. CD40L is constitutively highly expressed by a panoply of hematopoietic and non-hematopoietic cells [1,3,4,16]. CD40L can be further expressed or overexpressed by activated cells, the most characteristic and best studied of which are activated and/or differentiated T cells [4]. Like other members of the TNF family, active CD40L at the cell surface or in its soluble form is composed of homotrimers [17]. This multimeric conformation of CD40L is of crucial importance for effective interaction with CD40 and the subsequent intracellular signaling [18]. Moreover, the soluble forms of CD40L retain their ability to form trimers, which bind CD40 and deliver biological signals [18]. Membrane bound CD40L can be cleaved at methionine 113 of the extracellular domain and shed as a soluble form [19,20,21]. The principal isoform (isoform 1) is encoded by 5 exons. The second CD40L isoform (isoform 2) is poorly described (Figure 1). It is a truncated 240 AA protein lacking exon 4 in the *CD40LG* (extracellular domain), and the functional consequence of this is unknown [22]. Of note, membrane bound CD40L is expressed on B cells and dendritic cells (DCs). It is not expressed on non-activated T cells and platelets, but is weakly expressed on non-activated macrophages, neutrophils and endothelial cells [23]. It is highly expressed on activated T cells and platelets from which it can be cleaved as a soluble form, but it is not cleaved from B cells, DCs and macrophages. There is no up-regulation in neutrophils and endothelial cells, regardless of whether they are activated [1,3,4,23].

The main receptor for CD40L is CD40, which is constitutively expressed by antigen presenting cells (APCs) such as B cells, macrophages, and DCs [3,4,24]. CD40 is also expressed by platelets [25,26], neutrophils, endothelial cells [23] and T-cells [27,28,29,30]. Five distinct isoforms of CD40 are expressed with two isoforms predominating in human and mice [31,32,33]. Isoform 1 predominates and is membranous, but may be cleaved into a soluble form by a metalloproteinase, ADAM-17 [34]. In contrast, isoform 2 is produced as a soluble form resulting from alternative splicing [35]. It is hypothesized that the soluble forms act as competitive inhibitors for the membranous form, though this remains unclear [36]. Although CD40 is a type I TM protein that can form monomers, dimers and trimers, only the latter form fully activates cells [37,38,39].

CD40L can also bind to three integrins: the platelet glycoprotein αIIbβ3 (GPIIb/IIIa), otherwise known as receptor for fibrinogen and von Willebrand Factor [40,41]; α5β1 (CD49e/CD29), an integrin that binds to matrix macromolecules and proteinases and thereby stimulates angiogenesis [42,43,44]; and Mac-1, an integrin (otherwise known as CR3 (Complement Receptor 3), CD11b/CD18, or α_M_β2), mainly expressed by neutrophils, natural killer cells and macrophages to trigger a transduction signal and mediate inflammation [45]. The functional interaction of CD40L with α5β1 is independent of its binding to αIIbβ3 and CD40 [43,44]. Interactions between CD40L and α5β1 are not relevant in platelet physiology/physiopathology [45].

## 3. What Is the Function of CD40L?

The interaction between CD40 and CD40L is essential in the innate and adaptive immune systems, both in physiology and in physiopathology.

### 3.1. CD40/CD40L in Physiology

First characterized as a major marker on carcinoma cells, CD40 was next shown to be a key molecule shared by endothelial cells and most APCs, including B-cells, monocytes and DCs [46]. Interactions with CD40L are mandatory for the B-cell response to T-dependent Ags [2]. In particular, studies on patients with primary Ab immunodeficiencies targeting CD40 or CD40L have definitively established the requirement of these interactions for GC formation and the generation of memory B-cells and long-lived plasma cells [47]. More recent data on GC reactions and follicular helper T-cells (T_FH_) show that the polarization of CD4 T-cells into T_FH_ is initiated by contact with DCs at the border of B-cell follicles and maintained by GC B-cells [48]. The expression of BCL6, the master regulator of T_FH_, is dependent on CD40-CD40L and ICOS-ICOSL interactions outside follicles and within GCs [49]. CD40L-induced CD40 signaling in B-cells is crucial for inducing the expression of BCL6 and Ki67 in GC B-cells, allowing the proliferation of GC B-cells in the dark zone and expression of activation-induced deaminase (AID), a transcription factor required for somatic hypermutation (dark zone) and Ig class switching (light zone). CD40-CD40L interactions are further required for the selection of B-cell clones expressing high affinity BCR that takes place within the GC light zone. In physiological conditions, only selected B-cell clones differentiate into effector B-cells (memory and plasma cells). CD40 is also constitutively expressed by DCs and macrophages, and its triggering induces the expression of other co-stimulatory molecules and the release of cytokines that modulate T- and B-cell responses [24]. CD40 activation on macrophages also induces the release of nitric oxide and reactive oxygen species, contributing to the destruction of intracellular pathogens. Strikingly, CD40-induced CD40L signaling in CD8 T-cells rescues them from the exhaustion observed during chronic viral infections and is important to maintain their poly-functionality [50]. With CD40 being expressed on various B-cell lymphomas and carcinomas (nasopharynx, bladder, cervix, kidney and ovary), there is a renewed interest in CD40/CD40L in the control of tumor growth, leading to the development of new therapeutic strategies [51].

### 3.2. CD40L and Its Receptors in Inflammatory Pathologies

**Figure 2:**
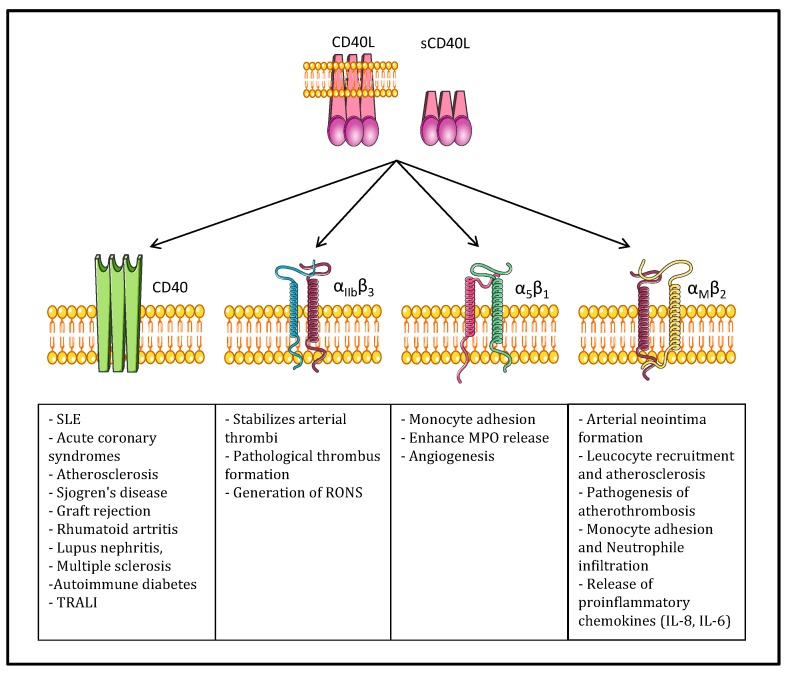
CD40L and its receptors: the binding of CD40L to CD40, αIIbβ3, α5β1, or Mac-1 (αMβ2) induces different inflammatory pathologies. Systemic lupus erythematosus (SLE), transfusion-related acute lung injury (TRALI), reactive oxygen and nitrogen species (RONS), Myeloperoxidase (MPO).

As already presented, in addition to the classical receptor CD40, CD40L also binds the αIIbβ3, α5β1, and Mac-1 (αMβ2) integrins and induces different biological responses. Figure 2 illustrates the pathological role of each dyad interaction.

The CD40-CD40L system is associated with both pro-thrombotic and pro-inflammatory effects. Soluble CD40L contributes to the pathophysiology of atherosclerosis and atherothrombosis [52]. Because of its autocrine, paracrine, and endocrine activities, sCD40L enhances platelet activation, aggregation, and platelet-leukocyte conjugation that may lead to atherothrombosis [13,53,54]. CD40L binding may result in the activation of CD40 expressing cells with interleukin production [23,55]. The interaction of CD40L with CD40 on endothelial and other vascular cells upregulates adhesion molecules such as E-selectin, VCAM-1, ICAM-1 and proinflammatory cytokines such as regulated on activation normal T cell expressed and secreted (RANTES), interleukin (IL)-6, and IL-8 as well as matrix metalloproteinase (MMP)-1, -2, -3, and -9 [56]. Soluble CD40L also stimulates the expression of tissue factor (TF) on monocytes and on endothelial cells [57,58]. After CD40L and CD40 interact on the endothelial surface, thrombomodulin expression is decreased, facilitating thrombin generation [59]. CD40L-CD40 interactions activate endothelial cells via either sCD40L *in vivo* or by a specific antibody to CD40. Membrane-bound CD40L, but not sCD40L, induces the upregulation of pro-inflammatory cytokines and cell adhesion factors in endothelial cells. However, both forms of CD40L activate both classical and alternative NF-κB pathways [60]. In addition, sCD40L induces endothelial dysfunction with decreased NO synthesis and augmented oxidative stress [61]. These events may further contribute to endothelium injury and accompanying atherogenesis. sCD40L may play a pathogenic role in triggering acute coronary syndromes [54,62]. The involvement of CD40-CD154 interactions in autoimmunity and allo-immunity is also well documented. In fact, many tissue injuries and immune mediated pathologies such as graft allo-rejections involve this signaling pathway [63]. CD40-CD40L interactions play a significant role in the production of auto-antibodies in systemic lupus erythematosus (SLE), rheumatoid arthritis (RA) and other autoimmune diseases. An increased serum level of soluble CD154 was reported in SLE, RA, and Sjogren’s disease, in correlation with the relevant auto-antibodies and with the clinical disease activity [14,64].

αIIbβ3 integrin was first identified as a receptor for CD40L by André *et al.* [65]. They showed that sCD40L can bind to αIIbβ3 integrin on activated platelets, thereby inducing platelet spreading and promoting platelet aggregation under high shear rates, as well as allowing stability of arterial thrombi [66]. The same group further reported that CD40L is a primary platelet agonist capable of inducing platelet activation, induction of fibrinogen binding and the formation of platelet microparticles by binding to its αIIbβ3 receptor and triggering outside-in signaling [67]. In addition, the engagement of αIIbβ3 by CD40L or other ligands that induce platelet adhesion upregulates CD40L surface exposure on platelets [68], enhancing the interaction of platelets with CD40+ cells, including ECs. Incubation of platelets with recombinant sCD40L led to enhanced P-selectin expression, aggregation, and platelet-leukocyte conjugation. The inhibition of either sCD40L or αIIbβ3 attenuated the generation of reactive oxygen and nitrogen species (RONS) by platelets [69].

Mac-1 is an important mediator of neutrophil and monocyte adhesion to the activated endothelium during inflammation. CD40L ligation to Mac-1 is involved in mediating CD40L/Mac-1-dependent monocyte and neutrophil adhesion and transmigration at the atherosclerotic lesion site, as well as neointimal formation during atherogenesis [42,70]. In transfusion, the sCD40L concentration increases in stored platelets compared to fresh platelets [9,10]. The neutrophil priming ability of stored platelets is significantly higher compared to fresh platelets [71]. Soluble CD40L and CD40-activated-neutrophils are essential to permit the adhesion and migration of neutrophils by Mac-1 secretion. This signal is the main system to recruit neutrophils into pulmonary tissue [72]. CD40+ neutrophils primed by CD40L+ activated platelets and sCD40L are recruited and over-stimulated by IL-6, IL-8 and IL-1β originating from alveolar macrophages and fibroblasts. In alveolar space, these neutrophils secrete ROS, proteases, PAF and elastase-α1-antitrypsin complexes that insult the pulmonary parenchyma [73]. In another study using the two-event TRALI mouse model, Hidalgo and colleagues demonstrated an increase in platelet interactions with adherent neutrophils in the systemic circulation [74]. These interactions were dependent on E-selectin expression on the endothelium interacting with E-selectin ligand on neutrophils, which ultimately led to the polarization of Mac-1 on the leading edge of the neutrophils. Circulating platelets interacted with the clustered Mac-1, although the platelet ligand mediating this interaction is not known [74]; could it be CD40L?

The α5β1 integrin is expressed by endothelial cells, smooth muscle cells, monocytes/macrophages and platelets. It is implicated in cell adhesion, migration, and proliferation as well as survival of many cell types. The binding of CD40L to a monocytic cell line expressing α5β1 integrin leads to the phosphorylation of the extracellular signal regulated kinases 1/2 (ERK-1/2) and expression of IL-8 mRNA in these cells [14]. However, unlike fibrinogen and vitronectin which are the natural ligands of α5β1, CD40L binds to the inactive rather than the active form of α5β1. Interestingly, CD40L/α5β1 interactions do not interfere with the binding of CD40L to CD40, indicating that CD40L can bind simultaneously to both receptors [43].

The role of α5β1 as a receptor for CD40L in α5β1-expressing-cells has not yet been investigated. Hassan *et al.* hypothesized the involvement of the CD40L/α5β1 dyad in angiogenesis and pathological conditions of the vascular system after the tethering of cells in inflamed tissues such as atherosclerotic lesion sites [75].

## 4. Platelet CD40L

**Figure 3:**
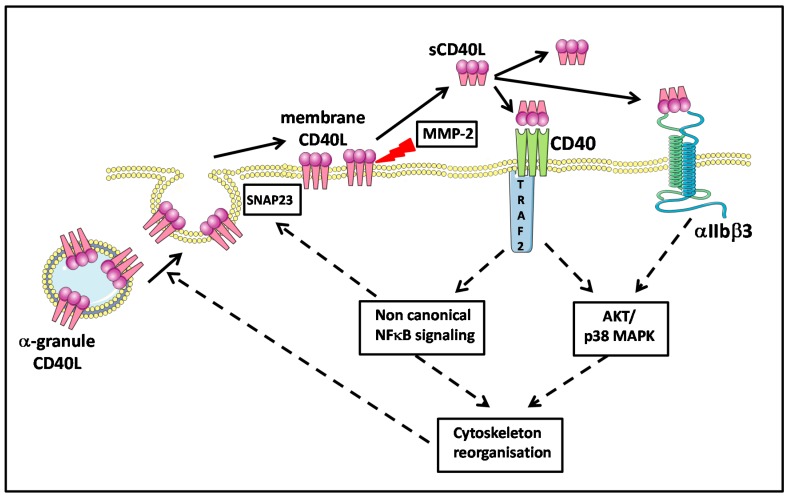
Schematic overview of the regulation of platelet CD40L and the role of sCD40L in signaling after binding to platelet CD40 and αIIbβ3 inducing an auto-amplification loop. Synaptosomal-associated protein 23 (SNAP23), mitogen-activated protein kinase (MAPK), nuclear factor kappa B (NF-κB), protein kinase B (AKT) matrix metalloproteinase-2 (MMP-2), TNF receptor associated factor 2 (TRAF2).

The discovery in 1998 that platelets preferentially express many copies of CD40L on their surfaces upon activation was surprising because CD40L was thought to characterize immune reactive cells, and platelets were not yet acknowledged to display any immune function [25]. CD40L was then found in platelet cytoplasm [25,65,76,77], and years later more precisely identified as being docked in the platelet α-granules [78] (Figure 3). The discovery that, despite being non-nucleated cells devoid of DNA apart from mitochondrial DNA [79], platelets can retrotranscribe RNA using a spliceosome [80,81,82,83] and lead to detectable RNA messages for cytokines, questioned the possibility that CD40L is also produced *de novo* by activated platelets. Recently, some RNA-seq studies did not find CD40L mRNA in platelets [84,85,86,87]. This result suggests that a preformed protein is synthesized by megakaryocytes and stored in α-granules before platelet fragmentation [88,89,90].

After stimulation by different agonists, platelets undergo a degranulation process via a well characterized mechanism [91], and either export the α-granule molecules to the membrane in a fixed form or secrete them as a soluble form. Granules fuse with the platelet membrane and display their fixed CD40L on the surface. This process occurs within seconds to minutes after stimulation [25]. CD40L is thus expressed on the platelet surface only after activation, and this molecule is identical in terms of structure and physiological function to membrane bound CD40L expressed in activated T-lymphocytes and other cells. It can notably generate signals for the recruitment and extravasation of leukocytes. It induces, through the engagement of CD40, the secretion of chemokines and the expression of adhesion receptors in endothelial cells [25]. It provides a powerful link between platelets and the immune system: CD40L expressed on activated platelets induces dendritic cell maturation, B-cell isotype switching, and augments CD8+ T-cell responses in both *in vitro* and *in vivo* models [92,93,94,95].

Platelets do not maintain CD40L on their surface for long. It is cleaved and released in a soluble form and may also be carried on the surface of microparticle-derived platelets [96]. Platelets are the major source of sCD40L in the circulation [65,97,98]. The normal range of sCD40L in the serum of a healthy adult is estimated at 0.79 to 4.7 ng/mL, by means of immunoassay techniques [99,100,101].

Of note, platelets constitutively express CD40 on their surfaces, both when resting and upon activation (Figure 3) [25,26,92]. This is surprising, as CD40 has long been considered to characterize APCs. Some sCD40L is reabsorbed on the platelet surface and principally binds CD40, a mechanism of recycling that must not be ignored when discussing platelet physiology and pathology.

## 5. Platelets, CD40L, and Molecular Signaling 

### 5.1. Platelet Activation in Platelet Components and Molecular Signaling

Platelet activation and the signaling pathways involved in hemostatic conditions are well documented [102,103,104]. However, there is little information regarding the platelet components (PCs) prepared and processed for transfusion.

Several proteomic studies have investigated platelet changes after either resting (*ex vivo*) conditions or stimulation (*in vitro*) [105,106]. Most have tested activation markers such as shape change, glycolysis, supernatant pH levels, platelet CD62P and CD40L surface expression, reactivity to repeated activation by agonists, secretion of platelet granule products, cytoskeletal reorganization and expression of apoptotic markers [9,107,108]. Most of those studies, as well as the subsequent ones, were carried out with the purpose of improving the platelet physiology in the *ex vivo* conditions that lead to the possibility of storing platelets for a limited number of days and transfusing homologous donor platelets to a recipient patient. The signaling pathways involved in the “spontaneous” activation of platelets in PCs were investigated [105,109,110].

Schubert *et al.* [109] found evidence for a signaling pathway mediating PC storage lesions in which PI3-kinase-dependent Rap1 activation leads to integrin αIIbβ3 activation and platelet degranulation. This pathway involves two principal actors: Rap1, a small GTPase that modulates αIIbβ3 affinity, most likely through effects on the actin cytoskeleton [111], and Talin, an adaptor protein that links αIIbβ3 to the actin cytoskeleton. In hemostasis, this pathway is activated by soluble molecules after binding to different receptors, leading to the activation of the integrin αIIbβ3 [112].

**Figure 4:**
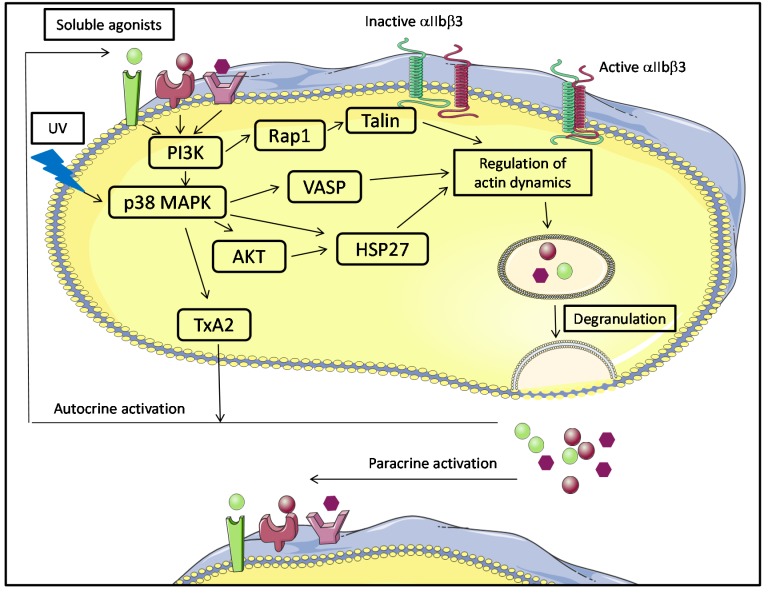
Principal signaling pathways inducing platelet activation in platelet components. Phosphoinositide 3-kinase (PI3K), mitogen-activated protein kinase (MAPK), protein kinase B (AKT), Thromboxane A2 (TxA2), Vasodilator-stimulated phosphoprotein (VASP), Ras-proximate-1 (Rap1), Heat shock protein 27 (HSP27).

Moreover, several studies identified the activation of the p38 MAPK signaling pathway during the aging of platelets not subjected to added stimulus [105,110], and/or after treatment of platelet concentrates with UV light with the intent of eradicating infectious pathogens. p38 MAPK is more highly activated after UV exposure, a PI3-kinase-dependent mechanism that involves AKT, VASP and HSP27. AKT thus acts as a substrate for p38 MAPK. HSP27 is a substrate for AKT, and it regulates actin dynamics and degranulation. This confirms the earlier finding that MAPK activation stimulates platelet degranulation and TxA2 synthesis, which may in turn activate platelets via the TP receptor [113]. After degranulation, soluble factors (ADP, ATP, TxA2, Ca^2+^ and thrombin) are released and may act quickly to amplify autocrine activation of platelets as well as the activation of surrounding platelets (Figure 4) [88].

Platelets possess a variety of pathogen recognition receptors (PRRs) to sense bacterial and viral moieties and other receptors that could be involved in platelet activation in PCs [114,115,116]. Activated platelets can, consequently, secrete inflammatory cytokines and chemokines and other biological response modifiers (BRMs), including sCD40L, which could be a good candidate for such autocrine activation loops in platelets (Figure 3 and Figure 4).

### 5.2. Platelet Membrane CD40L Regulation and Shedding

As already stated, CD40L was initially thought to be almost absent from the surface of non-activated platelets [117]. It has been recently reported, however, that resting platelets express very low levels of CD40L on their surface but can translocate massive amounts of CD40L to the surface within minutes of activation. This CD40L can subsequently be cleaved and released as a soluble molecule into the circulation [92,118,119]. Such CD40L would thus be mobilized from the α-granules (Figure 3).

Knowledge regarding CD40L cleavage, either from T-cells or from platelets, remains incomplete. Cleavage from T-cells has been attributed to two types of MMPs. Two other MMPs are also responsible for the cleavage of platelet membrane bound CD40L.

MMPs constitute a large family of more than 25 functionally related endopeptidases mediating the proteolytic cleavage of most matrix proteins, as well as several non-matrix proteins including cytokines, chemokines, adhesion molecules and surface receptors [120]. ADAM10 has been proposed as a candidate MMP for CD40L cleavage and shedding from T-cells [121]. ADAM17 has been shown to be another candidate in an *in vitro* model of Jurkat E6.1 T-cells, where the inhibition of both ADAM10 and ADAM17 nearly completely inhibited CD40L shedding from the cells, suggesting that no other MMP besides ADAM10 and ADAM17 is involved [122]. The mechanisms that cleave activated platelet CD40L appear to be quite different. Not only MMPs but also the integrin αIIbβ3 are mandatory. MMP2 is the best candidate, based on different experimental approaches [123,124,125,126]. A novel enzyme has revealed itself as a potential candidate, at least in pathological situations, as demonstrated in a mouse model of sepsis. Here, MMP9 was involved in the shedding of CD40L after platelet-neutrophil interaction. Again, different experimental approaches confirmed a role for MMP9 [127,128,129,130].

Interestingly, the enzymatic regulation of CD40L cleavage from CD40L-positive cells appears cell-dependent. Platelets and T-cells use different proteases to cleave sCD40L from their cell surfaces (MM2/MMP9, and ADAM10/ADAM17, respectively), despite both cell types containing all four identified enzymes. Among the possible explanations are the existence and particularities of the cytoplasmic or granule reservoirs. Those distinct mechanisms have functional consequences on signaling pathways triggered upon CD40L/CD40 activation between these two cell types.

In platelet CD40L cleavage, the need for functional and complete αIIbβ3 remains intriguing [123,131]. For example, Glanzmann thrombocytopenia patients fail to properly release sCD40L upon platelet activation [123].

### 5.3. Platelet and CD40L Signaling

CD40L production by platelets is an interesting intersection between hemostasis and inflammation. Hemostatic activation of platelets (by ADP, thrombin, collagen, *etc.*) induces inside-out signaling and, consequently, activation of αIIbβ3. This leads to outside-in signaling and degranulation, followed by CD40L expression on the membrane surface. After activation, CD40L is shed and released in an active form that can activate different cell types, including platelets.

Soluble CD40L may activate platelets via two independent receptors, CD40 and αIIbβ3. After sCD40L binding, both receptors activate AKT and enhance platelet p38 MAP kinase phosphorylation. One study showed that this signaling pathway initiates the generation of inflammatory molecules such as reactive oxygen and nitrogen species [69].

Soluble CD40L binding to platelet αIIbβ3 (through its KGD sequence) enhances thrombus formation and induces platelet spreading via outside-in integrin signaling in an auto-amplification loop [65,67]. This phenomenon also induces the generation of microparticles, especially through phosphorylation of tyrosine-759 in the cytoplasmic domain of the β3 chain [67].

Soluble CD40L may also activate platelets via the CD40 receptor, which is present on platelet membranes [25,26]. In this case, the mechanism is outside-in independent. The CD40L/CD40 activation in platelets involves a CD40-dependent TRAF2/Rac1/p38 MAPK signaling pathway and triggers phosphorylation of IkBα [132,133]. Thus, the sCD40L/CD40 interaction also triggers NF-κB pathway activation in platelets. In this case, NF-κB acts as a signaling molecule and not a transcription factor. IκB phosphorylates SNAP23, a key protein for the fusion of alpha granules and the plasma membrane [134]. IKKb blockade inhibits SNAP 23 phosphorylation and prevents SNARE complex formation (SNARE complex formation reviewed in [90,135,136]) and platelet degranulation [134]. These mechanisms are outlined in Figure 3.

## 6. CD40L and Platelet Component Transfusion 

The sCD40L association with platelets has been popularized because of the description of transfusion hazards [6,7,8,9,10,11,12]. Before that, although well published, this association received little consideration. For more than a decade, sCD40L-linked associated hazards also received modest consideration, probably because the attention of transfusiologists focused on preventable hazards, and residual leukocytes were considered to be responsible for all symptoms of inflammation [137]. Transfusion-linked inflammation was not yet acknowledged, but classed as discomfort. Accidents were attributed to other causes, which were sometimes reported as unidentified. Soluble CD40L gained attention when progress was made in the field of hemostasis and thrombosis, which outlined the role of platelets and leukocytes in the formation of atheroma plaque deposition and led to the proposal that cardiovascular disease is inflammatory [16,99,138,139,140,141,142,143].

Platelets in an inventory are generally stored no longer than 5 days (ranging from 3 to 7 days depending on country regulations). During storage, and without the addition of any stimulus intended to activate them, those so-called “resting” platelets are exposed to a number of stresses, including the process of constituting a PC, exposure to plastics, preservatives and gases, rotation, and changes in temperature [107,144,145]. Platelets are extremely reactive to external signals and are designed to sense external danger. They are equipped with many types of receptors and danger sensors, and they respond to multiple signals [83,116]. Anticoagulant factors and bacterial residues can modify the status of platelets that are believed to be “resting”, but which in fact are lightly stimulated just above physiological steady state [83,116]. As platelets secrete more pro-inflammatory than anti-inflammatory BRMs, they begin to produce or secrete BRMs that are fairly detectable in the PC supernatant by day 3 [9,146]. Soluble CD40L is the most visible cytokine-like BRM which is thus made, and it is produced in amounts that are sufficient to activate CD40+ cells *in vitro*, including B-cells, dendritic cells, and macrophages [146,147]. It is therefore fully bioactive. The longer the PC is stored, the more BRMs are found, apart from some molecules with extremely short half-life [11,12,146]. CD40L has a short half-life outside the α-granule, but its secretion over day 3, for 2 to 4 days, still allows biological function [146]. In general, PC transfusion is safe and accomplishes what it is expected to do: prevent or stop bleeding in the allocated patient/recipient. In approximately 10% of cases, moderate intolerance symptoms are reported, which are referred to as either febrile non-hemolytic transfusion reactions (FNHTRs) or allergic reactions (in fact, allergic-type reactions) [148]. In 2% of cases, the symptomatology is more severe, and presents more clearly as inflammatory [148]; such cases have been investigated by several groups, and there is a consensus on the responsibility of sCD40L that is found in excess in the PC or in the recipient’s plasma [6,7,10,11,12]. Soluble CD40L does not carry the full responsibility, but it is chiefly to blame [7,11,12]. It is also responsible in part for the physiopathology of a severe transfusion hazard called TRALI (Transfusion-Related Acute Lung Injury) [10], despite one recent publication that disputed this [149]. An open question is why some PCs seem loaded with sCD40L. If platelets in PCs can be over-stimulated by some unexpected event in the process, it probably does not occur in all cases [7,150].

## 7. Concluding Remarks: Towards Molecular Medicine Based upon CD40L and CD40 Polymorphisms

As the CD40/CD40L molecular tandem is essential in many pathways of physiological but also pathological immune and inflammatory responses, its control is valuable in patient care. We and other groups have worked extensively during the past few years on the involvement of sCD40L in transfusion associated hazards, and we have recently obtained evidence that there are a number of *CD40LG* polymorphisms that may affect the behavior of platelets in a PC processed for the purpose of transfusion [151]. Combined with polymorphisms of *CD40*, this may affect the preferential decrease of inhibitory isoforms of the molecules and the increase of high affinity isoforms. Certain platelet donors may express high levels of sCD40L that are promptly cleaved [11,12,126], and/or certain recipients express high affinity CD40 receptors on both circulating cells and endothelial cells, favoring excess CD40/CD40L reactions and adverse events. Cell signaling through these interactions may prompt those cells to either synthesize or release copious amounts of bioactive BRMS with inflammatory potential. If proven, donor selection and/or patient investigation would allow better matching to prevent such adverse events. Serious adverse events would also benefit from the recent development of biologicals that target either CD40 or CD40L. In fact, Tanaka *et al.* [152,153] have succeeded to remove 80% to 90% of sCD40L in PCs using a column of adsorptive cellulose beads. However, there was a significant decrease in the recovery of platelets after adsorption. In other diseases, blockade of CD40/CD40L was performed using anti-CD40L Abs, but unfortunately these drugs have exhibited potentially adverse interactions with platelets in patients [154].

Molecular or personalized medicine is thus underway for patients presenting with high risk of potentially lethal acute inflammatory responses. If not yet implementable at a large scale, this may be forecast for the very near future.
